# Reaching the Unreachable: Barriers of the Poorest to Accessing NGO Healthcare Services in Bangladesh

**Published:** 2006-12

**Authors:** Nizam U. Ahmed, Mohammed M. Alam, Fadia Sultana, Shahana N. Sayeed, Aliza M. Pressman, Mary Beth Powers

**Affiliations:** ^1^ Save the Children-USA, Bangladesh Country Office, House 1A(2), Road 91, Gulshan 2, Dhaka 1212, Bangladesh; ^2^ NGO Service Delivery Program, House NE(N)5, Road 88, Gulshan 2, Dhaka 1212; ^3^ NGP Service Delivery Program and (currently) Save the Children-USA, Bangladesh Country Office, House 1A(2), Road 91, Gulshan 2, Dhaka 1212; ^4^ NGO Service Delivery Program and (currently) Save the Children-USA, Bangladesh Country Office, House 1A(2), Road 91, Gulshan 2, Dhaka 1212; ^5^ Save the Children-USA, Westport, CT 06880, USA

**Keywords:** Health services, Healthcare, Poor, Perceptions, Bangladesh

## Abstract

The NGO Service Delivery Program (NSDP), a USAID-funded programme, is the largest NGO programme in Bangladesh. Its strategic flagship activity is the essential services package through which healthcare services are administered by NGOs in Bangladesh. The overall goal of the NSDP is to increase access to essential healthcare services by communities, especially the poor. Recognizing that the poorest in the community often have no access to essential healthcare services due to various barriers, a study was conducted to identify what the real barriers to access by the poor are. This included investigations to further understand the perceptions of the poor of real or imagined barriers to accessing healthcare; ways for healthcare centres to maximize services to the poor; how healthcare providers can maximize service-use; inter-personal communication between healthcare providers and those seeking healthcare among the poor; and ways to improve the capacity of service providers to reach the poorest segment of the community. The study, carried out in two phases, included 24 static and satellite clinics within the catchment areas of eight NGOs under the NSDP in Bangladesh, during June-September 2003. Participatory urban and rural appraisal techniques, focus-group discussions, and in-depth interviews were employed as research methods in the study. The target populations in the study included males and females, service-users and non-users, and special groups, such as fishermen, sex workers, potters, *Bedes* (river gypsies), and lower-caste people—all combined representing a heterogeneous community. The following four major categories of barriers emerged as roadblocks to accessing quality healthcare for the poor: (a) low income to be able to afford healthcare, (b) lack of awareness of the kind of healthcare services available, (c) deficiencies and inconsistencies in the quality of services, and (d) lack of close proximity to the healthcare facility. Those interviewed perceived their access problems to be: (a) a limited range of NGO services available as they felt what are available do not meet their demands; (b) a high service-charge for the healthcare services they sought; (c) higher prices of drugs at the facility compared to the market place; (d) a belief that the NGO clinics are primarily to serve the rich people, (e) lack of experienced doctors at the centres; and (f) the perception that the facility and its services were more oriented to women and children, but not to males. Others responded that they should be allowed to get treatment with credit and, if needed, payment should be waived for some due to their poverty level. While the results of the study revealed many perceptions of barriers to healthcare services by the poor, the feedback provided by the study indicates how important it is to learn from the poorest segment of society. This will assist healthcare providers and the healthcare system itself to become more sensitized to the needs and problems faced by this segment of the society and to make recommendations to remove barriers and improvement of access. Treatment with credit and waived payment for the poorest were also recommended as affordable alternative private healthcare services for the poor.

## INTRODUCTION

Although the overall maternal and child-health indicators of Bangladesh have improved dramatically over the past 25 years, neonatal, infant and maternal mortality still remains high. The problem is most often associated with poor people which includes 45% of the population of Bangladesh. (1) For example, the infant mortality rate has remained stagnant since 1999 at 65 per 1,000 livebirths (2). Among the poorest Bangladeshis, the rate is even higher at 90 per 1,000 livebirths (2). Maternal mortality in Bangladesh also remains high, with an unacceptable level of 300 deaths per every 100,000 livebirths (3). Contributing factor for the high levels of maternal and infant mortality is the lack of access to formal healthcare by poor women and their children for antenatal, childbirth, and postpartum services (2). Beyond the issue of poor access is the fact that people in this low-income group face added risk factors due to poor health in their daily living environment. In fact, the cause of 21.1% of deaths among Bangladeshi children aged less than five years in 2004 was acute respiratory infection (ARI) due to exposure to biomass fuel in an inadequately-ventilated home—a common feature of low-income households across South Asia (4). Efforts to prevent these deaths must, therefore, confront the poverty that contributes to their poor health.

In response to the alarming statistics above, the Government of Bangladesh has developed a detailed maternal and child-health policy framework that includes—as one major element—the essential services package (ESP), designed in 1997, for delivery of services at local clinics throughout the country. The services under the ESP include contraception, antenatal and postpartum care, diagnosis and treatment of sexually transmitted infections, treatment of tuberculosis, and therapy for ARIs and diarrhoea (3). The most recent Health, Nutrition and Population Sector Programme Implementation Plan of the Government stresses increasing accessibility of services for the poor; it recommends participatory meetings, lowering the barriers the poor face, and improving the targeting of services and health programmes for the poor (3).

Although the Government affirms the rights of all citizens to healthcare and officially endorses increased services for the poor, its own health system struggles with constant and evolving changes, problems with effectiveness of the healthcare-delivery system, and inadequate coverage of the population (5). Approximately, 30% of the population (National Health Audit Report, 2002–2003) is covered by government services. Those who can afford it typically use the services of private providers (5).

Perhaps in recognition of its own limitations, the Government now looks into the NGO sector to supplement public-health services. In fact, the Government has allowed and encouraged NGOs to provide key health services in specific geographical areas under-served by government facilities (6). The vibrant, indigenous NGO sector of Bangladesh has, thus, stepped up to the challenge and is playing a major role in health advances, particularly in managing tuberculosis, malnutrition, and neonatal mortality (6). A recent World Bank study notes that the grassroots approach carried out by NGOs makes them more effective in reaching the poor than public and private providers because they are locally based and are also more accountable to their communities (6).

The creation of the USAID-sponsored National Integrated Population and Health Programme (NIPHP) in 1997 can be seen as part of the trend towards supporting healthcare provision by NGOs. This strategy, endorsed by the Government, strengthens the Bangladeshi NGOs in their provision of the ESP. In 2002, USAID began funding the NGO Service Delivery Program (NSDP) through a consortium of eight international organizations. Technical support and guidance was offered through the NSDP to 37 Bangladeshi NGOs that administer a network of 318 static clinics and 7,814 satellite clinics, covering 20 million Bangladeshis in areas where government services are largely inaccessible. In addition to clinic-based services, many NGOs support female community workers, called depot-holders, who provide health commodities and make referrals to NGO clinics. A major expected outcome of the NSDP is that, due to improvements in the technical and managerial capacity, these NGOs will become self-sustaining after USAID funding ends. The NGO clinics of the NSDP charge for their services to promote cost-recovery and sustainability.

As the NSDP is mandated to increase service-use by the poor, partner NGOs and their accomplishments reflect a global shift in public-health services that not only address and attempt to solve inefficiency in access by the poor to healthcare services, but also demonstrate that injustice in allocation of health resources needs to be remedied worldwide for people in the economically-poor conditions similar to Bangladesh. (7). In a 2003 programme evaluation, despite its commitment to serving the least advantaged, the NSDP was found to have a limited effect on the health of the poorest quintile in project areas (8). The limitations are especially stark in child health: an evaluation of the child health initiatives of the NSDP estimated that the immunization coverage had declined in the NSDP areas between 2001 and 2003 and that the NSDP clinics were not significant sources of treatment for childhood ARI and diarrhoea (9).

To make services more responsive to the needs of the poor and, thereby, improve public health, the partner organizations of the NSDP—Save the Children-USA and CARE—provided institutional support to participatory formative research conducted during June-September 2003. This research obtained the opinions of the poor about their health conditions and the NSDP clinics that were available to serve them. It also looked into ways to remove barriers and improve relations between clinics and their respective communities. This paper presents the findings of that research, describes activities undertaken in response to the results of the research, and identifies the changes in the NSDP policy and service-use by the poor following these interventions.

The study was conducted to (a) identify perceptions and barriers among the poorest in accessing NGO healthcare services, (b) identify ways to maximize service-use by the poor, (c) enhance the interaction between community and NGO service providers, and (d) strengthen the capacity of service providers to reach the poorest in the community.

## MATERIALS AND METHODS

### Study design

Four rural and four urban NGOs participated in the formative research. The rural NGOs are: Jubo Unnayan Samaj Sheba Shangstha (JUSSS), Brahmanbaria, Swanirvar Bangladesh, Jamalpur, Jatiya Tarun Sangha (JTS), Rajshahi Debi Choudhurani Pally Unnayan Kendra (DCPUK), Kurigram, and the urban NGOs are: Family Development and Social Research (FDSR), Cox's Bazar, Concern Women for Family Development (CWFD), Dhaka, Sylhet Samaj Kalyan Sangstha (SSKS), Sylhet, and Fair Foundation, Khulna.

These NGOs were selected according to a geographical representation of the country. Recognizing the need to involve participants in communities where poverty most affects access to quality healthcare services, a participatory rural appraisal (PRA) approach was used throughout the research. In total, 101 PRA sessions were held, along with 73 focus-group discussions (FGDs) and 158 in-depth interviews (10). The results of the study include the categorization of poor and non-poor according to the own indicators of the communities and explanations of health-seeking behaviour as revealed in the health-mobility exercise. Participants also described a range of financial and non-financial barriers that prevented them from accessing NSDP services. This approach increased collaboration between community members and, in this case, NGO staff who facilitated sessions (11). FGDs with poor participants and in-depth interviews with a wide range of individuals were also applied. The study participants used both social mapping and wealth-ranking techniques for identifying the poorest among them. For the social mapping exercise, the participants drew maps of their villages to locate the poorest of the poor. In wealth-ranking sessions, the participants categorized the households in their neighbourhood as rich, middle-class, poor, and hardcore poor/poorest based on assets and income. Poor mothers with children aged less than five years were invited to participate in the health mobility mapping and Venn diagram exercises. The Venn diagram technique was used for exploring the perception about the NSDP clinic and the barriers in accessing the NSDP clinic by the poor and the poorest. Using this technique, the research team also tried to identify a set of ways and means to increase the use of services by the poorest segment of the population of the NSDP catchment areas.

In the health mobility-mapping activity, mothers of children aged less than five years described where and how far they went for their health needs, illustrating the role of proximity in health decisions. A Venn diagram exercise elicited perceptions of the NGO clinics and the barriers preventing participants from accessing services (10).

From the records of each sample clinic, two villages and slums were selected. From each selected village and slum, 200–300 households were covered after consulting the family register of the clinic. In selecting a study village and slum, the criterion of ‘poverty-stricken area’ was followed, i.e. of many villages in the project area, the most ‘poverty-stricken’ village and slum (according to the NGO staff and the members of the research team) were selected based on observation and transect walk. Both users and non-users of clinic services within these areas were targeted for participation in the study.

### Sample size and characteristics

Researchers invited villagers and slum-dwellers to participate in the research. In total, 627 people of both genders and a range of ages and income levels participated in PRA/participatory urban appraisal (PUA) meetings; 555 participated in FGDs. In each FGD, 10–12 poor and poorest of the poor participated. Seven in-depth interviews were also conducted under each clinic site. In total, 158 in-depth interviews were completed with 84 in rural areas and 74 in urban areas, and 588 spoke in in-depth interviews for a total of 1,770 community participants. The in-depth interviews were conducted with paramedics, depot-holders, opinion leaders, elected body members, non-user poorest females, user poorest females, and non-user poorest males.

The majority of the participants had one of three professions: 28.7% were daily labourers, 24.7% were housewives, and 20.7% worked in agriculture (Table 1). Members of other professions represented included hawkers, petty business-owners, self-employed, housemaids, and beggars (10). The in-depth interview participants included depot-holders, local elected officials, and prominent community members. Throughout the study, special attention was given to socially-marginalized groups, such as river gypsies, fishermen, sex workers, those of lower castes, and members of isolated communities (10).

**Table 1.** Percentage distribution of participants of urban and rural areas of eight NGO sites by age-group, education, and occupation status

Criteria	Number of participants	Percentage
Age-group (years)		
15–19	21	3.36
20–24	110	17.54
25–29	149	23.76
30–34	137	21.85
35–39	115	18.34
40–44	49	7.81
45–49	24	3.83
50+	22	3.51
Total	627	100.00
Educational status		
No education	219	35.00
Primary-level		
Class 1-V	**227**	**36.00**
Class VI-X	**125**	**20.00**
SSC	**39**	**6.00**
HSC and above	**17**	**3.00**
**Total**	**627**	**100.00**
Occupation (primary)		
Daily labour	180	28.71
Agriculture	130	20.73
Petty business	66	10.53
Ferry/hawker	**33**	**5.26**
Self-employed (sewing, traditional thin quilt)	**23**	**3.67**
Housemaid	**33**	**5.26**
Housewives	**155**	**24.72**
Beggars	**07**	**1.12**
Total	**627**	**100.00**

Source: NGO Service Delivery Program formative research, 2004

HSC=Higher Secondary School Certificate NGO=Non-governmental organization

SSC=Secondary School Certificate

## RESULTS

### Findings of participatory rural appraisal

The findings of the PRA revealed a widespread feeling among poor women who had no adequate access to healthcare and faced discrimination at most types of facilities. The poorest women preferred to go to the government facilities because they could receive medicines and services free of charge. Most rural PRA participants stated that services in the healthcare facilities depended a lot on nepotism and favouritism and that discrimination against the poor was widespread. Some urban respondents stated that service-delivery of NGOs was beneficial to them as poor. Some important findings are summarized below:

- Poor women received more health services from unqualified doctors, *kabiraj*, homeopaths, traditional healers, Upazila Health Complex, registered doctors, and government hospitals than from the NSDP clinics.
- According to the participants, the satellite clinics provided immunization for children and family-planning commodities. Packets of oral rehydration solution were available with depot-holders.
- Most PRA participants lived within one km of the location of homeopaths and traditional healers and received healthcare services from them.
- Personal relationships played an important role in receiving services from the NSDP clinics. Well-off families had a good relationship with the NSDP clinic staff and received better healthcare services than the poor.
- The participants expected to receive essential services free of charge as the Government does not formally charge users for most primary healthcare services.
- Health services should be more accessible to poor women, and NGO health providers should be accountable to the poor.

### Findings of focus-group discussions and in-depth interviews

During FGDs and in-depth interviews, the participants shared their comments and suggestions spontaneously. The results of FGDs and in-depth interviews were, on balance, consistent with those of the PRA exercises. Some key responses elicited during FGDs and in-depth interviews are given below;

- Quality of treatment at the NSDP clinics was good. Doctors listened to patients, and their behaviour was good.
- Prices of medicines were cheaper compared to market prices.
- Waiting space was comfortable, and patients did not have to wait for a long time.
- It was a good organization for health services, but the process of treatment was very slow.
- Family planning, treatment of pregnant women, and child healthcare were provided in the clinic.
- Satellite clinics provided treatment for general diseases.
- Prices of medicines were higher than those of the open market
- Healthcare services were not available for men.
- There were no qualified doctors. Health service providers were inexperienced.
- The government health facility provided services free of charge, but the NSDP clinic charged service-fee.
- Privacy of clients was maintained in the clinic.
- The community people expected medicines from the NSDP clinics at a reduced rate.

### Wealth ranking by participants

Both rural and urban participants mentioned perceptions of and barriers to accessing the NSDP clinics, however, the nature of statement differed between the rural and the urban participants. The study participants ranked those in their communities along a four-tier scale: rich, middle, poor, and poorest. The results of the wealth-ranking exercise were not, however, comparable with national demographic data because they were gathered in the specially-selected sites in a participatory, non-scientific manner. The indicators used for defining poverty varied from location to location, reflecting the diverse experience of poverty in Bangladesh. According to a composite of rural and urban PRA sessions, 35% of the rural study population was categorized as poor, and 30% was categorized as the poorest (Table 2). In urban slums, 21% of the study population was identified as poor, and 45% was classified as the poorest class (10).

**Table 2.** Percentage distribution of rich, middle, poor, and poorest by eight sampled rural and urban NGOs

Class	Rural NGOs	Urban NGOs
Rich	10.00	11.35
Middle	25.00	22.38
Poor	35.00	21.17
Poorest	30.00	45.10
Total	100.00	100.00

Source: NGO Service Delivery Program formative research, 2004

### Health-seeking behaviours

In the health mobility exercise, poor mothers of children aged less than five years discussed where and how far they went for services. Distance for services played a major role in healthcare-seeking behaviour of rural participants as time spent travelling represented a significant opportunity cost, especially for household wage-earners. Travel was also expensive, increasing the real cost of healthcare. All the rural participants in this exercise had seen an unqualified village doctor or a *kabiraj*, a type of spiritual healer, who could be found within one km of their residence. Traditional healers, pharmacies (run by unqualified pharmacists), and family welfare clinics were between 1 and 5 km away from their home and enjoyed the patronage of 60% of the participants. Several participants noted their appreciation of deferred payment options and customer-friendly environment of pharmacies (10).

The rural participants used the formal health system, albeit less frequently than informal providers. Fifty percent of the village participants had visited doctors with MBBS degree (1–5 km away) or the Upazila Health Complex (administrative district) 1–16 km from their home. The participants preferred the Upazilla Health Complex because it offers a range of free treatments, hospitalization is free, and it includes three meals a day. Between 20% and 50% of the participants visited the NGO clinics other than the NSDP clinics, which were 3–5 km away. The participants reported that the depot-holder was less than one km away, but she was ranked tenth among 11 types of facilities in terms of frequency of visit. This may be because of limited range of services that the depot-holder offers (10).

For urban poor women, proximity was less of a challenge, and health-seeking behaviour differed greatly. The most popular choice for healthcare was a local quack, found 1–3 km or less from their home. The second choice was an NGO clinic, which was attended by 70% of the participants. District Sadar Hospitals, or government hospitals, were the third choice and were 1–8 km away. Although *kabiraj* could be found nearby, they were the seventh most popular choice in urban areas, opposed to the third choice in rural areas (10). The urban women benefited from having competing health options close by. It is disconcerting, however, that their preferred option is still an unlicensed health provider.

### Perceptions of financial barriers

It is not surprising that poor participants would consider cost a major constraint to their healthcare choices. Since the government health services are free, many participants felt that it was unethical and exploitive for the NGO clinics to charge for the same services. Some participants expected that the services of the NSDP-affiliated NGO clinics would be free because they are ‘run by foreign aid’ (10). Several participants also had the impression that the NSDP-affiliated NGOs charged higher than market prices for their drugs. The FGDs recorded diverse impressions, including that the poor did not use the NSDP clinics, and, in contrast to the above, that medicines in the NSDP clinics were available at below market prices. To better serve the poor, participants suggested that NSDP clinics exempt the poorest from service-charges and provide them with free medicines. They believed that the NGOs should be more flexible when dealing with the poor and accept deferred payment from those who cannot provide the service-fee at the time of visit (10).

### Perceptions of quality treatment offered by service providers

Opinions about service providers were elicited during the Venn diagram exercises. Discussions of the quality of the NSDP clinics revealed that the poor did not use a strictly clinical standard to define the quality of health services. Instead, they placed a high priority on being treated with respect and in a timely manner. Many participants believed that the rich received preferential treatment at the NSDP clinics, while the poor were treated with disrespect. Some participants believed that patients were treated well at the NSDP clinics and that ‘privacy was maintained’ (10).

Perceptions of quality identified in FGDs in urban areas and in in-depth interviews differed from the results of PRA. In FGDs, the urban respondents thought that the quality of the NSDP clinics was high, doctors listened to patients, and paramedics were capable. However, they stated that treatment was very slow and that the poor were neglected compared to the rich. The findings of in-depth interviews on the quality issue varied widely, perhaps due to the socioeconomic diversity of those interviewed. Some stated that doctors were not qualified, medicines did not work, and services providers were disrespectful with clients. However, others had a more positive impression saying that ‘treatment for children was satisfactory’, and service providers were good (10).

### Perceptions of quality of clinical facilities and availability of services

Although many participants did not seek formal healthcare on a regular basis, they did voice complaints about the clinical standards at the NSDP clinics. Several complained that doctors were not qualified or that paramedics (who staff satellite clinics) were inferior to MBBS doctors (10).

Some critics of the quality of services reflected the needs of the poor mentioning that the NSDP clinics were ill-equipped to meet their needs. A recurrent comment was that the NSDP clinics did not treat all illnesses. This is true; the NSDP clinics have limited mandates and resources. They are not open at all hours, which limits their use for emergency care. Rural participants suggested that all medical services should be provided at the NSDP clinics, while urban participants requested that the clinics be open at all hours. Others requested ambulances for emergencies (10). Although the NSDP clinics are not presently designed to meet these demands, these suggestions offered policy-makers a guide on how to better meet the needs of the poor.

Comments of women in the health mobility exercise on the availability of health services indicated that poor women wanted to have one convenient provider for their healthcare needs rather than be obliged to use a host of different providers for treatment and referrals. Those services that treat all types of diseases, are conveniently located, and are available at the customer's convenience, were the most popular among the participants. Interestingly, those facilities regarded as having a good standard of treatment did not receive a great deal of business from participants (10).

### Perceptions of information about healthcare and availability of services

Lack of information about the NGO clinics was perceived as a major barrier. The fact that many participants believed that the NSDP-affiliated NGO clinics were private clinics for the rich indicates that previous outreach efforts were not successful in informing the poor about the NSDP clinics. One participant commented that the poor did not know about the NGO clinics because the clinic staff did not visit them. The FGDs revealed similar results with one rural participant noting that the ‘communication of the NGOs system was bad’ (10).

The PRA participants had a wealth of suggestions to overcome the information barrier. Some urban participants suggested advertising NGO clinics through the media, using television, films, and posters. One urban participant suggested the distribution of free t-shirts with the Smiling Sun logo (brand identity of NSDP). In rural areas, many participants preferred a grassroots approach. Groups of the poor who could meet clinic staff could then promote clinic use in their communities. Local clubs and voluntary associations could also be mobilized. Some believed that clinic staff should visit the poor and tell them about clinics, while others thought that announcements through mosque loudspeakers would help lower the information barrier (10).

### Restitution (discussions of results between providers and study participants)

At the conclusion of the formative research, results were shared with the study population in participatory restitution meetings. The service providers responded to criticisms and explained the limitations preventing them from fulfilling all the suggestions of the participants. The study participants and service providers discussed concrete steps to address the barriers in the research and to continue the dialogue between the poor clients and the service providers. Following restitution meetings, the eight NGOs, together with community members, developed detailed action plans to overcome the barriers identified in the research. This level of cooperation was entirely new and represented a shift in dynamics between the clinic staff and the community members towards greater appreciation and partnership.

## DISCUSSION

### Interventions envisioned to remove barriers

The results of formative research offered the NSDP and its partner NGOs a roadmap for removing barriers and attracting poor customers to its clinics. Following the research, the community response team helped NGOs scale up participatory approaches. Local-level groups that provide the interface between the communities and the service providers were reorganized; partnership-oriented quality tools were introduced; and a positive deviance inquiry was introduced. The results of formative research also spurred more pro-poor programming across the NSDP and helped change the project policy.

Prior to formative research, many NSDP-affiliated NGOs were associated with community organizations, called satellite clinic support groups (SCSGs) and static clinic advisory teams (SCATs). These groups were meant to network with clinics and improve the quality of services. After formative research, these groups were reorganized and became mobilized on behalf of the community. The NSDP designed a new scope of work for these groups, requiring them to include at least one extremely poor community member. The SCSGs and SCATs were given a clear goal: to increase the use of services by the poorest of the poor. Under this mandate, the SCSGs and SCATs have raised thousands of taka to underwrite the health services of the poorest. Membership in these organizations has increased to 90,000 across all the NSDP clinic sites.

One of the most important post-formative research interventions was the official introduction of partnership-defined quality (PDQ), a methodology developed by Save the Children-USA in 2004. As the formative research demonstrates, a gulf lies between the way of service providers and clients, especially poor clients, define quality. The PDQ method facilitates cooperation between the service providers and the community to improve the quality of services from the perspectives of both service providers and consumers. In the PDQ process, a facilitator elicits the definitions of service quality of both providers and clients and encourages both the parties to share perspectives and prioritize key quality concerns. Groups, called Clinic-Community Quality Teams, are formed which work to ensure the satisfaction of both clinic providers and community members with services of their NGO clinics. The Community Response Team devised a new PDQ manual, specifically for the NSDP-affiliated NGOs, which incorporated the feedback of clinic staff. The NSDP trained 683 NGO staff members in the PDQ techniques during capacity-building workshops. Twenty-four clinics, corresponding to 10 NGOs, have introduced PDQ; in these locations, poor community members have been involved at every step of the process.

In an effort to improve health-seeking behaviour among the poor, the NSDP partner—Save the Children-USA—introduced its positive deviance inquiry (PDI) approach in impoverished areas. Positive deviants are those who defy social norms and pursue healthy behaviours, such as seeking antenatal care. The approach is based on the notion that advising the poor on healthy behaviour is not always enough; many people need to see an example in their own, difficult environment to change. The community is involved at all steps of the PDI process. In participatory meetings, community members identify a reproductive or child health issue they believe is important and name a positive deviant (PD) in their community who practises this behaviour. Through a series of meetings, the individual adhering to the ideal practice speaks about why she chose to deviate from her peers and answers questions from other women in the community. As the meeting attendees live close to one another, the PD is also available to speak to her neighbours at their convenience. This approach both reinforces and promotes positive behaviour and uses local role models to whom other community members can relate. The PDI method increases collaboration between clinic staff and community members and encourages the poor community members to consider using clinic services. All NGO clinic managers and service providers have been trained in the PDI methods, and 32 clinics of 11 NGOs have now begun the PDI process.

The efforts of the community response team to increase service-use by the poor are complemented by an increased focus on the poor across the NSDP programme areas. One of these initiatives is a creative mystery client approach in which service providers attend distant NGO clinics under NSDP dressed as a poor person and then share their impressions with clinic staff. The 2004 NSDP communications strategy addresses the communication barrier the poor face and encourages local-level interpersonal communication efforts, which are more likely to reach the poor than national media campaigns as the poor have less access to media than wealthy people (2). The NSDP has also developed a television drama serial promoting its clinics that encourages viewers to support increasing access to health services for the poor.

### Addressing the financial barrier

The formative research and efforts to identify the poorest of the poor illustrated that the cost of services was a severe obstacle for the poor. To alleviate this burden, several financial measures were devised that are now piloting at several NSDP sites. One of the most promising methods is the health benefit card that was distributed in areas where the poorest of the poor have been identified. The card entitles the holder to free access to the core health services offered by the NSDP-affiliated NGOs. The least advantaged (LA) who are new customers pay a registration fee of Tk 5 (US$ 0.8) in urban areas and Tk 2 (US$ 0.3) in rural areas.

In 2004, the NSDP and its partner NGOs developed a pricing and exemption policy to balance obligations of NGOs to serve the poorest of the poor with the imperative of financial sustainability. The policy offered five different options to NGOs ranging from soliciting *zakat*, or charity, to creating a pricing structure that would subsidize free services to those clients who can least afford them.

To further encourage NGOs to serve the poor, the NSDP is piloting an incentive scheme in four NGOs. Under this scheme, the NSDP will reward NGOs for their performance in serving the poorest of the poor by reimbursing them for the income they would have earned from paying-clients. This policy answers the need of NGOs to be compensated for their efforts to reach the poorest of the poor. Even with the guidance of NSDP, managing the cost of outreach to the destitute is challenging for NGOs, which often lack financial sophistication.

### Corporate social responsibility and serving the poor

Corporate support of the NSDP clinics in the form of corporate social responsibility (CSR) benefits the poor both directly and indirectly. Direct benefits accrue through sponsored health services, and an indirect benefit is that, by enhancing institutional sustainability, CSR increases the likelihood that the Smiling Sun clinics will provide services to the poor after the USAID funding ends. One notable example of CSR is British-American Tobacco, Bangladesh's (BATB's) sponsorship of the Smiling Sun clinics in areas where its farmers live. The BATB pays for the care that farmers and their families receive at the Smiling Sun clinics, salaries of doctors, and 15% of clinic overhead costs. The BATB further demonstrated its commitment to employees by financing the expansion of Smiling Sun satellite clinics to remote, tobacco-growing areas. The multinational UNOCAL provides support to the Smiling Sun clinics under an agreement reached in April 2005 to fund a new static clinic near its oil-field in Nabiganj. The NSDP-affiliated NGOs have also forged relationships with the garments industry, promoting workplace initiatives in 62 factories. The Standard Chartered Bank branched out into a new area of CSR by sponsoring the airtime for a Smiling Sun clinic commercial, starring Mohammed Rafique, a renowned Bangladeshi cricket player.

### Policy changes

The outcome of the formative research has influenced the development of NSDP policy. After reviewing the study, the NSDP leadership introduced a new pricing policy designed to better meet the needs of the poor. Although it was and is NSDP policy not to turn any customer away because of her inability to pay, in fact, not all the clinics adhered to this policy, and it was not effective in attracting poor customers. To increase service-use by the poor, the NSDP introduced the health benefit card, which allows those identified as the poorest of the poor to have a consultation at any NSDP clinic free of charge (12). The NSDP policy does not, however, cover the cost of prescriptions for the poorest of the poor. The cards were introduced in April 2004, and the number and percentage of poor customers attending the NSDP clinics have increased since then.

Other pricing policy changes occurred following the formative research. Several NGOs introduced a deferred payment system, as the study participants requested, while others altered their pricing policies to reduce costs for the poor and simultaneously promote cost-recovery (13). The NSDP also developed five options for financing services to the poor, including the use of profits from the sale of drugs, allocating a percentage of total revenue towards service to the poor, raising money through *zakat* (charity), and selling old equipment. Although balancing service to the poorest of the poor with cost-recovery remains a challenge for NGOs, these policy changes suggest that the NSDP has adopted a more pro-poor posture.

### Effect on service-use

The number of poor customers served by the NSDP clinics has risen since the formative research. These data are self-reported by NGOs and used by the NSDP for programme monitoring. Interpretation is limited by the fact that the definition of ‘poor’ changed in March 2003 following a major effort to identify poor families within catchment area of each clinic. Many NGOs, however, continued to report those clients as ‘poor’ who were unable to pay, whether or not they were classified as non-poor by the NSDP. Nevertheless, a trend can be detected from the overall project data (Fig.). The percentage of customers considered to be poor has more than doubled since the inception of outreach efforts. The major change came in the spring of 2004 when pro-poor initiatives scaled up. The trend has, however, remained somewhat flat around 19% of all users since August 2004. In January 2003, only 7% of all clients were poor; the percentage has now reached 18.9%. The number of poor clients served has more than tripled during that period, from 111,461 in January 2003 to 343,057 in June 2005.

**Fig.**
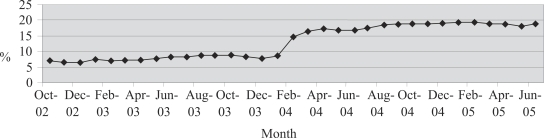
Trend of service-use by poor customers

Source: Management information system data of NGO Service Delivery Program, 2005

The results of the formative research were quite similar to those of a qualitative study pursued in the catchment areas of USAID-supported NGOs in 1998 and 2000 documented by Schuler *et al.* (14). Common themes among poor subjects included low inclination to pay for health services, except in the case of emergencies, and complaints about treatment by service providers. In both the studies, poor participants/respondents objected to having to pay for health services at the NGO clinics because the government services were free. Schuler *et al*. recorded that poor respondents felt it unethical to charge them service-fees because the Government should be responsible for their care (14). The study also revealed that poor customers viewed the number of pills they receive as a measure of quality; therefore, a rich woman who receives more pills receives superior service. The similarity between complaints over time and in different locations suggests that the poor have a deep distrust on healthcare providers. Overcoming this wariness will take time, but the NSDP's promotion of dialogue between the poor and service providers seems a logical and much-needed step.

In the afore-mentioned study and in the formative research, cost represented a significant barrier for the poor. Distance, which contributes to the real cost of services, has been documented as a significant barrier for the poor in Bangladesh and India in previous studies (15, 16). Even the decision to use any form of contraception, which has been found not to be price-sensitive in a previous study, can be influenced by the distance of the provider (17). The afore-mentioned Indian study by Griffiths and Stephenson found that distance and cost barriers do not restrict the use of antenatal care when women know the benefits of the service (15). In that case, poor women learned about antenatal care from a community health worker (15). The NSDP has a network of depot-holders who could perhaps be more involved in education and outreach. Supervision and quality control are an issue. The NSDP uses a pro-poor communications strategy that operates at a local level. The extent to which these messages reach the poorest and most isolated has not been evaluated, however.

The formative research gave voice to the opinions and needs of the poor. The pro-poor interventions that followed are built upon the research, underscoring the value of community mobilization in reaching the poor. The poorest members of Bangladesh society distrust authority and place a low priority on their health. Increasing use of service among this population is only possible once trust has been built, and the barriers of cost, distance, and misinformation have been lowered. Through efforts like PDI, the poor learn that they can change their behaviour, if not their surroundings, and have better health outcomes. This, in turn, may generate demand for formal health services from the poor. The increase in NGO service-use by the poor following the introduction of a partnership approach and the implementation of pro-poor pricing strategies suggest that involving the poor in their own healthcare is an important and effective way to promote health equity.

The participatory efforts that the NSDP continues to pursue represent a valuable way to promote sustainability. When a community embraces an institution, it is more likely to continue. Now that NGOs have learnt the participatory techniques, NGOs must integrate them into their day-to-day management. The SCSGs and SCATs should be more active and promote linkages with other community groups and religious organizations. They are a vital link with communities and can contribute more than they are at present.

In addition to their commitment to the poor, the NSDP-affiliated NGOs should work towards technical and financial sustainability. The balance NGOs should strike between cost-recovery and outreach to the poor is delicate and influences their commitment to health equity. Relying solely on cost-recovery from user-fees will not ensure the sustainability of NGOs in rural, poor areas where the majority cannot pay. These NGOs should look beyond pricing schemes and enlist the help of wealthy donors or forge partnerships with corporate sponsors. They should also lobby the Government for support in fulfilling the Government's health agenda. If NGOs in poor areas rely strictly on cost-recovery through user-fees, they will inevitably stray from their goal of service to the poor. Balancing both financial sustainability and service to the poor, therefore, requires NGOs to be both creative and committed to serving the poor.

Pro-poor policies and programmes are still scaling up, and their long-term impact is unknown. Overcoming the poor's wariness of formal healthcare providers and transforming attitudes of service providers will take time; these changes cannot occur overnight. It seems, however, that partner NGOs have made a strong start, and with continued commitment, they will continue to make a difference in the health of the poorest Bangladeshis.

## ACKNOWLEDGEMENTS

The authors acknowledge the contribution and support of the Chief of Party of NSDP Mr. Robert J. Timmons and Deputy Chief of Party Ms Tamara Smith, technical partner organizations of the NSDP, the Community Response Team of NSDP, all staff members of NSDP, partner NGOs, project participants, Prof. Abul Barkat and his team of the Human Development Research Centre, Government of Bangladesh, especially Ministry of Health and Family Welfare, Health, Population and Nutrition Sector team of Save the Children-USA, Bangladesh Country Office and head office at Washington, DC, and USAID Bangladesh.
